# *Drosophila melanogaster* as an Alternative Model to Higher Organisms for In Vivo Lung Research

**DOI:** 10.3390/ijms251910324

**Published:** 2024-09-25

**Authors:** Birte Ehrhardt, Thomas Roeder, Susanne Krauss-Etschmann

**Affiliations:** 1 Division of Early Life Origins of Chronic Lung Diseases, Research Center Borstel-Leibniz Lung Center, Airway Research Center North (ARCN), German Center for Lung Research (DZL), 23845 Borstel, Germany; 2 Division of Molecular Physiology, Institute of Zoology, Christian-Albrechts University Kiel, Airway Research Center North (ARCN), German Center for Lung Research (DZL), 24118 Kiel, Germany; 3 DZL Laboratory for Experimental Microbiome Research, Research Center Borstel, Airway Research Center North (ARCN), German Center for Lung Research (DZL), 23845 Borstel, Germany; 4 Institute of Experimental Medicine, Christian-Albrechts-University Kiel, 24105 Kiel, Germany

**Keywords:** *Drosophila melanogaster*, COPD, asthma, lung research, alternative model

## Abstract

COPD and asthma are lung diseases that cause considerable burden to more than 800 million people worldwide. As both lung diseases are so far incurable, it is mandatory to understand the mechanisms underlying disease development and progression for developing novel therapeutic approaches. Exposures to environmental cues such as cigarette smoke in earliest life are known to increase disease risks in the individual’s own future. To explore the pathomechanisms leading to later airway disease, mammalian models are instrumental. However, such in vivo experiments are time-consuming and burdensome for the animals, which applies in particular to transgenerational studies. Along this line, the fruit fly *Drosophila melanogaster* comes with several advantages for research in this field. The short lifespan facilitates transgenerational studies. A high number of evolutionary conserved signaling pathways, together with a large toolbox for tissue-specific gene modification, has the potential to identify novel target genes involved in disease development. A well-defined airway microbiome could help to untangle interactions between disease development and microbiome composition. In the following article, *Drosophila melanogaster* is therefore presented and discussed as an alternative in vivo model to investigate airway diseases that can complement and/or replace models in higher organisms.

## 1. Introduction

Chronic obstructive pulmonary disease (COPD) and asthma are by far the most common respiratory diseases worldwide. With around 800 million patients, the number of affected persons exceeds the total population of the EU member states, which illustrates the tremendous burden for the people and consequently healthcare systems [1,2].

**Figure 1:**
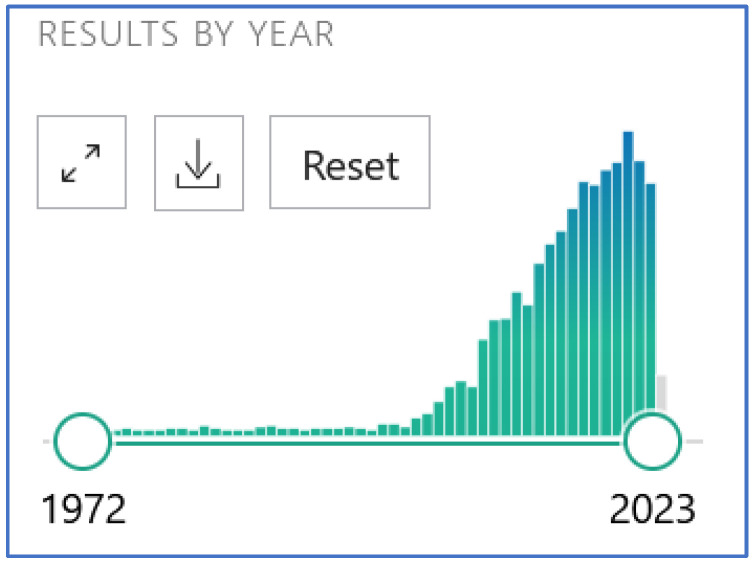
Publications on experimental COPD in mice from 1972 to 2023.

COPD is a progressive disease characterized by irreversible alveolar destruction and chronic bronchitis, each to variable degrees. By causing more than three million deaths per year worldwide, it is currently the third most common cause of death after coronary heart disease and ischemic strokes [3]. In addition to the clinical symptoms of cough, shortness of breath, and sputum production, the diagnosis is made based on impaired lung function. The number of COPD patients is expected to continue to rise in the coming years, partly due to the aging population. The disease only occurs in later adulthood, with cigarette smoking—followed by air pollutants and occupational exposure to toxic dusts—being the most potent risk factor for the development of the disease. As COPD is incurable, considerable efforts are being made to identify new therapeutic targets and to better understand the pathophysiology with the help of animal models. This is illustrated in Figure 1, which shows the frequency distribution of publications on experimental COPD exclusively in mice, without reviews (search term in PubMed “COPD Mouse models NOT reviews”), and yielded 2047 hits. Despite a slight decrease in 2022 and 2023, the high number and increase of experiments on COPD in mammals is evident.

An important risk factor for COPD is harmful environmental influence in early childhood, which prevents normal lung growth [4,5]. Epidemiological studies have provided clear evidence that early childhood exposure to tobacco products is significantly associated with the risk of developing COPD many decades later [4,6,7,8]. This critical early window of life includes the prenatal phase, as maternal smoking during pregnancy is an established risk factor for impaired lung health in offspring. Moreover, even preconception smoking by future parents may anchor epigenetic changes in the gametes that are transmissible to the next generation [9]. The prepuberty of later fathers is particularly important here, as during this time primordial germ cells mature into spermatogonia, which in turn are long-lived stem cells to produce mature spermatozoa in the adult male. In other words, epigenetic changes that are rooted in spermatogonia will also be found in the resulting mature spermatocytes and can be transferred to the oocyte, where they can influence first transcriptional programs in the embryo [10].

Nonetheless, the underlying mechanisms of how disease risks are established in early life or even during preconception are still poorly understood and are therefore currently being investigated in mammalian models that are both lengthy and may conflict with animal welfare. This has prompted us to look for alternative methods that can provide a better understanding of the mechanisms underlying COPD development, with the ultimate goal of developing new therapeutic approaches for this disease.

*Drosophila melanogaster* is an insect with a life cycle of around 10 days at 25 °C. This life cycle includes four developmental stages: egg (embryo), larva, pupa, and adult. Embryonic development in the egg usually takes one day. The hatching larva is referred to as L1 larva. During larval development, the L1 larva grows to L2 and L3 larva in the time course of about 5 days at 25 °C. Late L3 larvae wander outside the medium to find a spot for pupation. During the pupal stage, a complete metamorphosis takes place, where the larval body is deconstructed and reorganized. After approximately 4 days, the adult ecloses from the pupa [11]. 

*Drosophila melanogaster* appeared as a particularly attractive alternative in vivo model to study cigarette-smoke-induced lung diseases such as COPD for the reasons detailed below. Since risk factors for COPD can take effect very early on, but the disease itself only manifests decades later, the fruit fly as a substitute for mammalian models of COPD has clear advantages for the field of study: (1) The short life span of approximately 90 days allows the observation of the animals throughout the entire life cycle, from early developmental stages to the adult fly. (2) *D. melanogaster* develops extracorporeally so that all developmental stages can be observed easily. (3) Like the human respiratory tract, the respiratory system of the fruit fly consists of hierarchically arranged branches that end in primitive alveolar-like structures in the fly [12]. (4) As in humans, the airway epithelium consists of a physical and immunological barrier [13,14]. (5) Like all inhaled pollutants, smoke first hits the surface epithelium of the lungs. In fruit flies too, the airways consist exclusively of epithelial cells. Similar to human airways, their epithelial cells comprise different subtypes [15]. (6) *D. melanogaster* has homologues to approximately 80% of all human disease-associated genes [16,17,18]. (7) The tissue-specific controlled overexpression or knockdown of such genes is very easy in *D. melanogaster* compared to mammalian models [19]. This also applies to fluorochrome-labeled reporter lines for the visualization of modified genes in the airway tract. (8) Regarding COPD, there were already indications from our work in *D. melanogaster* that cigarette smoke reduces body fat and increases metabolic turnover [20], as in COPD patients, and also disrupts airway development [21]. Additionally, exposure of female virgin flies to e-nicotine (as the first experiment on e-cigarettes) significantly reduced the growth of their offspring from larvae through pupation to adult flies [22].

However, the fruit fly has developed strong physical and physiological protective mechanisms as it feeds on decomposing plants and, therefore, needs to minimize the risk of infection. To prevent pathogens and toxins from entering its respiratory tract, the fruit fly can pause its breathing for prolonged periods [23]. It was, therefore, previously unknown whether cigarette smoke enters the respiratory tract or if it is predominantly absorbed via the body surface. The clarification of this question is an essential prerequisite for the widespread use of fruit flies as an alternative method for COPD studies in mammals. In the following, we therefore compare the conventional mouse models with the new *Drosophila* model to discuss the advantages and disadvantages of the new model. For that purpose, we focus on COPD research.

Additionally, we provide an outlook for the use of the alternative models in other fields of respiratory research, such as asthma and cancer models, and for the investigation of new exposure systems, such as E-cigarettes.

## 2. Mouse Models of Cigarette-Smoke-Induced Early Life Lung Diseases

**Figure 2:**
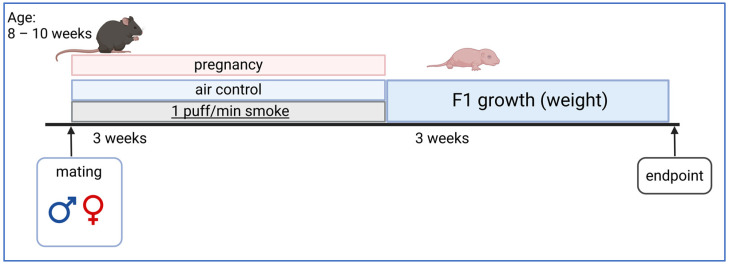
Scheme of in utero exposure in the mammalian mouse model. Males and females are mated at the age of 8 to 10 weeks. During pregnancy, females are exposed to cigarette smoke. The gestation period is 3 weeks and the rearing of the young is a further 3 weeks. Created with BioRender.com, last accessed on 23 September 2024.

**Figure 3:**
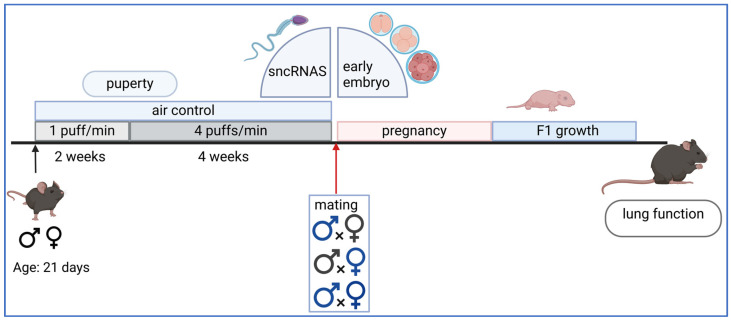
Model of pre-conceptional smoke exposure in the mammalian mouse model. Mice reach puberty at around 4 weeks of age and are considered adults at 8 weeks. The gestation period of a mouse is 3 weeks and the rearing of the young takes a further 3 weeks. A lung function test was carried out at 6 weeks of age. Figure adapted from: Hammer, B., L. Kadalayil [30]. Created with BioRender.com, last accessed 31 May 2024.

Inter- or transgenerational mouse models are used to investigate how exposure to risk factors, even in early childhood, causes the later manifestation of diseases such as COPD in adulthood [9,24]. It is distinguished between in utero exposure via the pregnant female (Figure 2) on the one hand and pre-conceptional exposure on the other (Figure 3).

For exposure during pregnancy, female animals are usually co-housed with males for 24 h for mating and then exposed to cigarette smoke. Standardized cigarettes (1R6F Research Cigarettes; Tobacco Research Institute, University of Kentucky, Lexington, KY, USA) are used for this purpose. No sedation is necessary for the exposure, and the animals move freely in the chamber of the exposure system (SCIREQ, Montréal, QC, Canada). After an adaptation period with a low cigarette dose (1 puff/min), the dose is increased up to 4 puffs/min, which corresponds to a heavy smoker dose (Figure 2). Between cigarette puffs, the animals are supplied with fresh air. The standardized exposure is objectified by particle measurements, measurement of Cyp1a1, and cotinine in serum or urine. Female animals only exposed to room air in an exposure chamber serve as a control group. As pregnancies cannot be reliably detected using the so-called plug test (it gives only an indication that copulation has taken place but not successful fertilization), approx. 70% of the females drop out during the experiment. In offspring from the remaining 30% of the animals, the effects of in utero exposure on birth weight, growth, lung function, molecular parameters, epigenetics, and immune functions, including resistance to infection or asthma risk, are investigated [25,26,27,28,29].

Two-generation models in mammals are used to elucidate the underlying mechanisms of pre-conceptual cigarette smoke exposure. For this purpose, female and male mice are separately exposed to a low dose of cigarette smoke (1 puff/min) at the age of 21 days and then to a higher dose (4 puffs/min) for 4 weeks. Afterward, mating with the opposite-sex partner takes place, and gametes, as well as offspring, are investigated (Figure 3). So far, the paternal sperm have been examined for changes in non-coding small RNAs (sncRNAs) [30], as well as the transcriptome of the first embryonic stages, which could be influenced by the altered sncRNAs in sperm (unpublished data). In addition, the offspring’s growth and lung function can be examined. The progeny of parental pairs in which the sire and dam were not smoked serve as controls.

## 3. Induction of Lung Disease in the Adult Mammalian Model

Mouse strains are predominantly used for the experimental induction of COPD in adult animals, but also (less frequently) hamsters [31], rat strains, and guinea pigs [32]. There are two different methods for the induction of experimental COPD in mammalian models [33]: The older but still used [34,35] method involves intratracheal instillation of porcine pancreatic elastase or human neutrophil elastase under anesthesia into the lung. After enzyme instillation, the animals initially develop severe pneumonia with weight loss, which over the course of three weeks results in an emphysema-like destruction of the lung tissue. In the second method, which is closer to the human situation, mice are exposed to cigarette smoke, which probably better reflects the pathophysiology of COPD than the intratracheal instillation of an enzyme. In this model, mice are usually exposed to cigarette smoke every day, except weekends, for 6 to 9 months to produce a COPD-like emphysema. The daily duration differs depending on the model but is usually not less than one hour per day.

## 4. *Drosophila melanogaster* as an Alternative In Vivo Model for Studying Cigarette-Smoke-Induced Respiratory Diseases

To establish the fruit fly as a suitable alternative in vivo model for early life cigarette-smoke-induced respiratory diseases, such as COPD, it was first necessary to assess whether smoke reaches the fly’s respiratory tract when exposed to cigarette smoke. As mentioned above, the fly can survive without breathing for a longer time to protect itself from pathogen invasion. In humans, cigarette smoke activates the aryl-hydrocarbon receptor (AHR) in bronchial epithelial cells. This receptor then translocates to the nucleus, where it binds together with the AHR nuclear translocator (ARNT) to the dioxin-responsive element to induce the transcription of the cytochrome P450 family gene Cyp1A1, which is required for the degradation of polycyclic aromatic hydrocarbons present in cigarette smoke [36]. We demonstrated that cigarette smoke activates a gene of the cytochrome P450 family called *Cyp18A1* in the airway epithelial cells of fruit flies [37]. This molecule is homologous to human Cyp1A1. For the first time, we showed that cigarette smoke is absorbed via the respiratory tract of the fruit fly larva and distributed through it. The fruit fly, therefore, proved suitable as an alternative in vivo model.

The pathobiology of respiratory diseases such as COPD is characterized by inflammation and tissue damage in the lung epithelium. These are caused, among other factors, by the body’s disturbed antioxidant defense mechanisms [38,39]. Further susceptibility to COPD development has been reported to be higher through polymorphisms in oxidative stress response genes, such as GSTM1 [40] or heat shock proteins [41,42]. We therefore visualized the stress response in airway epithelial cells in situ using the diverse genetic tools characteristically available in fruit flies. For this purpose, a transgenic fly line expressing the green fluorescent protein GFP under the transcriptional control of heat shock protein 70 (*hsp70*) was applied. In response to cigarette smoke exposure, a clear GFP signal could be measured in the epithelial cells of the entire airways, thus proving that smoke indeed enters the main conducting airway of the fruit fly [37].

To further prove and characterize the epithelial response to cigarette smoke exposure in early development, airways were isolated from larvae, and gene expression was analyzed by RNA sequencing. With this analysis, we provided firm evidence that, similar to humans [43,44,45], cigarette smoke causes a strong change in the transcriptional response of the respiratory tissue. Interestingly, there was a clear sex-specific difference in the transcriptional response to cigarette smoke exposure [37]. To understand the transcript changes in more detail, we performed a gene ontology analysis. This revealed that genes of the oxidative stress response in particular, as well as the FGF signaling pathway, the innate immune response, and G-protein coupled receptor signaling (GPCR) pathways, were differentially expressed. However, the genes encoding pathway components were often sex-specific and up- or downregulated in opposite directions. For example, genes of the GPCR signaling pathway and innate immune response were upregulated in females, whereas they were downregulated in males. Some signaling pathways were specifically altered in females, including genes of the Notch signaling pathway or genes of the Wnt signaling pathway. Other signaling pathways were exclusively altered in males, including xenobiotic metabolism genes and the BMP signaling pathway [37].

To validate the results of the RNA sequencing, gene expression was tested by quantitative qRT-PCR. The analysis focused on antagonists of oxidative stress [37]. Again, a clear difference between males and females was observed, with gene expression in males often being much higher than in females, suggesting that oxidative stress was greater in male airways, possibly due to their smaller body size. To detect and quantify oxidative stress in the airways, reactive oxygen species were detected and quantified in the airways [37].

To analyze the influence of cigarette smoke exposure in early life on further development, exposed larvae and air controls were followed during their further development and until natural death. Similar to humans, cigarette smoke exposure in the larval stage led to increased larval mortality in males. Similarly, the metamorphosis rate was reduced in males, meaning that fewer males could complete their development into adult flies [37]. The lifespan and oviposition ability of the adults were not altered [37].

To test if *Drosophila* is also a suitable model for the investigation of cigarette smoke exposure in adulthood, we confronted adult flies with different exposure regimens. Interestingly, the life span was dramatically reduced, even by short exposure periods of 30 min per day [20]. The measurement of body fat levels showed a strong reduction in body fat content of females after 7 days of exposure. Additionally, resting metabolic rates were nearly twice as high in cigarette-smoke-exposed flies as in the controls [20]. Moreover, daily doses of cigarette smoke led to a strong reduction in overall physical activity. Here, males were already strongly affected after 7 days of exposure, while effects on the daily activity in females were only visible after 14 days of exposure [20]. Altogether, the features seen in the fruit fly mirror clinical and metabolic characteristics seen in adult COPD patients.

**Figure 4:**
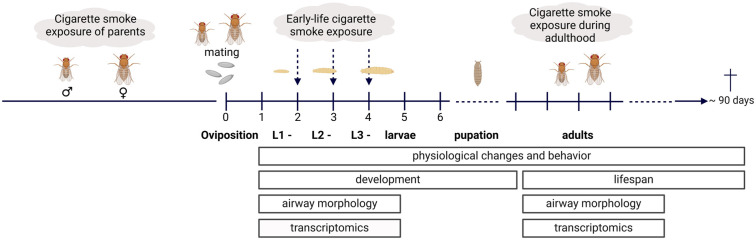
Overview of the alternative in vivo model and possible applications. Cigarette smoke exposure is possible in parents as well as in early developmental stages. Furthermore, exposure can only occur in adult life. Possible study parameters are morphological airway changes, genetic changes, or changes in the transcriptome, as well as physiological effects on behavior, development, and lifespan. Created with BioRender.com, last accessed 31 May 2024.

Taken together, these experiments established the fruit fly as a suitable alternative in vivo model for studying cigarette-smoke-induced respiratory diseases such as COPD (Table 1). In particular, the possibility of investigating the responses of the airway epithelium in the early stages of development is a great benefit, as this is hardly possible in humans for ethical reasons and induces burden in higher model organisms. The comparatively short life span of the fruit fly also makes it possible to track pheno- and endotypes over the entire lifetime (Figure 4) and also (in future experiments) to observe them over several generations. Another advantage is that the differences between the sexes can be studied easily and effectively. Furthermore, we could show that the transcriptional response to cigarette smoke in the airway epithelium is comparable to the situation in humans. Many altered signaling pathways and genes have already been linked to the pathogenesis of inflammatory lung diseases or are involved in lung development. When exposed to cigarette smoke in adult life, *Drosophila* shows a set of pathological phenotypes, which are also seen in the human situation, especially in COPD patients [1]. Worth mentioning here is a strong reduction in life span, accompanied by reduced levels of physical activity, increased metabolic rates, and reduced body fat content. This once again underlines the suitability of the fruit fly model in airway research.

## 5. Discussion

With the establishment of the alternative in vivo model for cigarette-smoke-induced respiratory diseases, such as COPD, we have laid the foundation for further research in this area while at the same time reducing the number of mammalian animals required. Our fruit fly model is particularly suitable for investigating cigarette-smoke-induced changes in the airway epithelium. Our smoke exposure system for *D. melanogaster* is identical to that used for mice, ensuring a rigorous and comparable experimental setup. Since the metabolism of the fruit fly differs significantly from that of the mouse, the indirect adjustment of the exposure is carried out via the increase in the cytochrome P450 gene *Cyp18a1* by qPCR in the smoke group relative to the air group. *Cyp18a1* is homologous to CYP1a1 in mammals, giving an approximate estimate of the exposures.

Every stage, from embryonic to adult exposure, is comparatively easy to investigate. Sampling is possible at any stage of development. A study spanning several generations requires much less time as compared to mammalian models, which last, depending on the readouts and sampling timepoints as well as the duration of cigarette smoke exposure, between four months and up to one year per generation under study (Table 1). If animals should be investigated until natural death, experiments last up to two years. The fruit fly has a clear advantage when it comes to monitoring lifespan, as this is only around 90 days in the fruit fly (Figure 4). Both morphological and genetic changes can be investigated. In addition, physiological effects, e.g., on behavior, lifespan, or developmental time, can be observed without requiring extensive instrument equipment. For example, larval fitness can be measured with the crawling assay [54], while adult fitness and activity can be assessed with the geotaxis assay [54] or by using *Drosophila* activity measurement systems [55].

One of the key strengths of fruit flies as a genetic model, which has been utilized for many decades, is the vast number of genetic tools at our disposal. Moreover, it is comparatively easy to produce and study genetically modified organisms (GMOs) in fruit flies. The Gal4-UAS system allows us to induce tissue-specific overexpression or knockdown of the genes of interest [19]. Thus, it is also a potential application of our model to investigate possible risk genes (Table 2) and the interaction of these with external factors (such as cigarette smoke) in the airway epithelium [21,56]. In addition, it is also feasible to identify new risk genes via genetic screens and investigate them specifically in fruit flies. Conversely, rescue experiments can also be carried out on the fly.

The fruit fly can also be used for immunological studies [14,60]. However, the focus here is on the innate system, as the fruit fly does not have an adaptive immune system. Nevertheless, infection experiments can also be carried out in the fly [13,60]. Investigation of epigenetic changes in the fruit fly involves the analysis of histone modifications and non-coding RNAs, as DNA methylation does not occur.

Since fruit flies have a less diverse respiratory microbiome than mammals [61], studies on the effect of genetic changes on the composition of the respiratory microbiome are conceivable. These could serve to broadly investigate questions of interaction between microbiome, exposures, and genetics and then reduce them to more specific questions that can only be answered in mammals or cell culture systems.

As with every model system, the fruit fly also has some limitations. Unlike in mammals, airway function cannot be directly assessed in fruit flies. However, an increase in lactate dehydrogenase (LDH), which indirectly reflects tissue hypoxia, can be visualized in vivo by using the corresponding LDH-reporter lines. Further, a respirometry test can be used to indirectly measure oxygen consumption by measuring the CO_2_ emission of the fruit fly [62]. These tests provide information on the basal metabolic rates, which are physiologically linked to respiratory functioning. Lung function parameters, such as lung elasticity, airway resistance, and lung volume, however, must still be measured in the mouse. For a detailed comparison of humans, mice, and fruit flies, see Table 1.

As mentioned above, the fruit fly relies exclusively on an innate immune system and has an open blood circulation system in which the so-called hemolymph circulates. Accordingly, adaptive immune responses cannot be investigated in fruit flies.

The eggs and larvae of fruit flies grow extracorporeally, such that the influence of a shared environment of the mother and her in utero developing offspring cannot be investigated. However, the eggs in the fruit fly are also fertilized within the female tract before they are laid. Therefore, the direct influence of the mother on the development of the offspring exists at the time of fertilization, which is then propagated indirectly during embryonic development. On the other hand, the extracorporeal development of the embryo allows a detailed view of the entire development, which has led to *Drosophila* being a popular model for embryonic development, leading to groundbreaking findings that laid the foundation for our understanding of developmental processes today [11].

## 6. Outlook: Other Potential Applications in Respiratory Research

The fruit fly represents a new form of model organism for the field of respiratory diseases. The model is already being used beyond the field of COPD. This offers further opportunities to also combine the smoking model of *Drosophila* presented here with research in other respiratory disease areas. 

### 6.1. Cigarette-Smoke-Associated Disease

Lung cancer is the leading cause of cancer-related deaths worldwide, and cigarette smoke is the leading cause of lung cancer [63]. In recent years, Bossen, J., K. Uliczka [64] developed a *Drosophila* lung cancer model to investigate oncogenic driver mutations of non-small cell lung cancers, particularly for the oncogene *EGFR*. They could show that targeted ectopic expression of a constitutively active isoform of *EGFR* in the airway epithelium of flies led to excessive tissue growth, leading to early larval death. Moreover, they used the model in a high-throughput screening approach to identify potential compounds rescuing this phenotype and thus identify potential therapeutic targets [64]. It is a thrilling idea to combine their lung cancer model with the cigarette smoke exposure model presented here to investigate mechanisms of lung cancer development in the *Drosophila* model system [64].

### 6.2. Cigarette Smoke Replacement

Electronic cigarettes are advertised as healthier tools for smoking cessation, especially for pregnant women [65], but it is not clear how vaping might affect future offspring. Therefore, we developed a *Drosophila* model for e-nicotine exposure [22] as the first step towards e-vape exposure. In a transgenerational model, we then investigated airway health from the offspring of exposed mothers. We found that airway morphology in the offspring of vaping mothers was altered. Furthermore, general fitness was reduced in the offspring until adulthood [22]. These data underline the need for epidemiological studies on the long-term effects of vaping devices. We now aim to further develop our system to investigate the effects of e-cigarette vapor. With this system, we will then be able to also mimic a smoking cessation model by combining our *Drosophila* smoking model and the vaping model to investigate the effects on offspring health.

### 6.3. Non-Cigarette-Smoke-Dependent Diseases

Asthma is the most common chronic lung disease in children worldwide. Asthma affects 1–29% of the population, with the number of cases continuing to rise, especially in children of second-world countries [2]. Clinically, asthma manifests itself through coughing episodes, shortness of breath, and mucus production. This is caused by airway hypersensitivity and chronic airway inflammation, which can also persist in asymptomatic phases [2,50]. Despite extensive scientific research, the disease remains incurable [66]. It is therefore still essential to further investigate the mechanisms of asthma development to gain a better understanding of the disease and potential new approaches for prevention. The risk of developing asthma in early life is shaped by various factors. Early life cigarette smoke exposure is a well-recognized risk factor for the later development of asthma. Additionally, gene polymorphisms within certain genes have been associated with asthma development. As risk factors are suspected to also interact with each other, the *Drosophila* model provides a suitable platform to investigate such interactions in the airway epithelium. The airway epithelium plays a central role in asthma pathogenesis [67], which is difficult to investigate in mammalian models because of the interplay with other tissues. For the most famous asthma risk gene polymorphism ORMDL3, Kallsen, K., N. Zehethofer [21] could show that overexpression of the gene in the airways of *Drosophila* larvae changes airway morphology, fitness, and the transcriptome dramatically. Thus, flies with altered gene expression were not able to handle exposure to hypoxic conditions anymore. Furthermore, they found that the cellular ability to respond to stressors adequately was abolished in the airway epithelium after the overexpression of the risk gene. Additionally, the repair capacities of the epithelium were reduced substantially [21]. They concluded that these changes in cellular response characteristics might reflect changes in the human situation, which then promote asthma development. In an ongoing project, we combined this powerful genetic model with our early life exposure model to investigate interactions between the two factors [56].

In summary, the *Drosophila* model offers the unique opportunity to investigate solely airway epithelial responses and physiological consequences without the interaction of various other cell types, as is the case in the mammalian situation. This is ideal for studying molecular changes ultimately leading to airway remodeling.

## 7. Conclusions

In the present work, we have laid the foundation for further use of the alternative model in COPD, asthma, and respiratory research.

Our *Drosophila* model is of great value to the field of respiratory research, as it helps to reduce the use of mammalian model animals in line with the 3R principles (reduce, refine, replace). Therefore, we propose the fruit fly as a relevant and attractive alternative in vivo model in COPD research. Additionally, our smoking model can be combined with other disease models to study cigarette-smoke-associated diseases, such as lung cancer, or to investigate the effects of cigarette smoke replacement. Further, non-cigarette-smoke-dependent diseases, such as asthma, might be investigated. This will help to uncover novel evolutionary conserved targets in early disease development, better understand the disease’s developmental origins, and find therapeutic solutions. In addition, the *Drosophila* model can be used to investigate promising signaling pathways and mechanisms to carry out a precise pre-selection of targets for further investigation in human cell culture systems, mammalian models, and ultimately human clinical trials.

## Figures and Tables

**Table 1:** Comparison of the various described readouts in humans, mice, and fruit flies.

		Human	Mouse	Fruit Fly
**General features**	**Lifespan**	~80 years	~2 years	~90 days
	**Body size**	Males > females	Males < females	Males < females
	**Chromosomes (n)**	46	20	4; giant chromosomes in some organs
	**Genes**	~20,000 protein coding [46]	~20,000 protein coding [47]	~14,000 protein coding [48]
	**Immune system**	Innate and adaptive	Innate and adaptive	Innate
	**Tissue-specific gene manipulation**	No	Complex	Fast and easy
**Respiratory system**	**Breathing**	Active via diaphragm and intercostal muscles	Active via diaphragm and intercostal muscles	Passive via body (larvae) or wing (adult fly) movements
	**Airways**	23–26 generations of branching; cartilage rings [49]	13 generations of branching [49]	3 generations of branching [12]
	**Airway** **epithelium**	Physical and immunological barrier, built from 8 different cell populations [49,50]	Physical and immunological barrier, built from 8 different cell populations [49]	Larval airways: Physical and immunological barrier, built as single—layered epithelium of airway epithelial cells and other cell types, such as neuroendocrine cells (unpublished data [51]). Pupal airways: nine cell clusters, two cell populations with multipotency [52]
	**Lung** **parenchyma**	2 lobes on the left and 3 on the right	1 lobe on the left and 4 on the right	No lung parenchyma
	**Gas exchange**	Via alveoli and perialveolar capillary bed [49]	Via alveoli and perialveolar capillary bed [49]	Passive diffusion into surrounding tissues via alveolar like structures (terminal cells) [53]
	**Lung function testing**	Used in diagnostics	Detailed analysis, invasive as endpoint. Non-invasive possible, but less informative	Only indirect measurement possible
	**Respiratory** **microbiome**	Complex	Complex	Few genera
	**Visualization**	CT, Bronchoscopy, Microscopy of biopsies	CT, Bronchoscopy, Histological slices of lung tissue	Micro CT; Microscopy of full body or isolated tracheae (several staining techniques available)
**Cigarette smoke exposure**	**Early life**	Indirect epidemiological assessment	Difficult to separate from maternal exposure	Possible via larval exposure
	**Prenatal life**	Indirect epidemiological assessment via smoking mother	Feasible but time-consuming via mothers	Exposure of embryos in eggs (fast)
	**Smoke exposure of pubescent**	Indirect epidemiological assessment	Possible but time-consuming	Virgin adults or Pupae (as equivalent to rapid hormonal and morphological changes)
**Inter—Transgenerational studies**	**Embryonic development**	In utero	In utero	extracorporeal
	**Generations needed to be transgenerational**	3 (maternal)	3 (maternal)	2 (maternal)
	**Epigenetic** **machinery**	DNA methylation, Histone modifications, non-coding RNAs	DNA methylation, Histone modifications, non-coding RNAs	Histone modifications and non-coding RNAs
	**Generation time**	20–30 years	~12 weeks	10–12 days (25 °C)

**Table 2:** Selected human COPD risk genes and their respective orthologues in mice and fruit flies.

Human COPD Risk Gene (Reviewed by Silverman, E.K. [57])	Mouse Orthologue [58,59]	Fly Orthologue [58,59]
AAT	Aatk	*Aatf*
ADAMTSL3	Adamtsl3	*nolo*
ADCY5	Adcy5	CG43373
ARNTL	Bmal1	-
ASAP2	Asap2	*Asap*
AGER	AGER	-
BTC	Btc	-
C1orf87	Gm12695	-
CCDC69	Ccdc69	-
CHRNA3	Chrna3	*nAChRα3*
IREB2	Ireb2	*Irp-1A*
CHRNA5	Chrna5	*nAChRβ2*
CITED2	Cited2	-
COL15A1	Col15a1	*Mp*
CYP2A6	Cyp2a5	*phtm*
DDX1	Ddx1	*Ddx1*
DENND2D	Dennd2d	-
DLC1	Dlc1	*cv-c*
DTWD1	Dtwd1	*CG2006*
EML4	Eml4	*DCX-EMAP*
FAM13A	FAM13A	*CG6424*
FBLN5	FBLN5	-
FGF18	Fgf18	-
HHIP region	HHIP	-
HSPA4	Hspa4	*Hsp110*
ID4	Id4	*emc*
IER3	Ier3	*CG32069*
IREB2	IREB2	*Irp-1A*
ITGB8	Itgb8	*Itgbn*
MFHAS1	Mfhas1	-
MMP1	MMP1	Mmp1
MMP12	MMP12	-
MTCL1	Mtcl1	*CG18304*
PPT2 region	Ppt2	*Ppt2*
PTPRO	Ptpro	-
RFX6	Rfx6	-
RIN3	Rin3	*spri*
RREB1	Rreb1	*peb*
SERP2	Serp2	*CG32276*
SERPINA1	Serpina1a	*Spn43Ad* *Spn28Dc*
SERPINA1 Z	Serpina1a Serpina1d Serpina1e Serpina1c Serpina1b Serpina1f	*Spn28Dc*
SERPINA6	Serpina6	*Spn28Dc*
SFTPD	Sftpd	*CG15358*
SLMAP	Slmap	*Slmap*
SNRPF	Snrpf	*SmF*
STN1	Stn1	-
TEPP	Spmip8	-
TERT	Tert	-
TGFB2 locus	Tgfb2	-
THRA	Thra	-
VGLL4	Vgll4	*Tgi*

## Data Availability

No new data were created or analyzed in this study.
